# Editorial for Special Issue “Advances in Soil Microbiome”

**DOI:** 10.3390/microorganisms11082026

**Published:** 2023-08-07

**Authors:** Ryan McClure

**Affiliations:** Biological Sciences Division, Pacific Northwest National Laboratory, Richland, WA 99352, USA; ryan.mcclure@pnnl.gov

The soil microbiome (the community of all soil microorganisms and their surrounding environment) is a critical part of our ecological network. These microbial communities carry out several important functions, including the promotion of plant growth and the cycling of carbon and nitrogen sources. Despite the importance of this community, and the recent advances in multi-omic analysis and other methods designed to query complex systems, there is a still a large knowledge gap regarding the soil microbiome, its response to changing climates and moisture levels, the specific molecular mechanisms that drive its role in the environment, and how these questions can be answered through the use of model systems. Here, we present eleven new papers that seek to answer these questions and expand our knowledge of the soil microbiome.

In “Soil Metabolomics Predict Microbial Taxa as Biomarkers of Moisture Status in Soils from a Tidal Wetland”, Dr. RoyChowdhury et al. tested how soil communities would respond to extreme wetting conditions [1]. This analysis was not limited to taxonomy but also included a deep analysis of the metabolomic profile of the soil, tying their results not only to presence or absence of certain species but also to the metabolic phenotypes of these communities. The authors took another major step by testing the power of the metabolomic profile to predict which taxa might be present. This opened the door to not only a better understanding of how moisture affects soil taxonomy and metabolite profiles but also how certain kinds of -omics data sets can be linked to others.

Continuing the analysis of water driving the soil microbiome, Dr. Ren et al. examined how water levels (closer to or higher than the current water table of the Poyang Lake wetlands) affected microbial diversity as well as levels of total nitrogen, recombinant organic carbon, particle organic carbon, and microbial biomass carbon (“Water Level Has Higher Influence on Soil Organic Carbon and Microbial Community in Poyang Lake Wetland Than Vegetation Type”) [2]. All of these measurements were found to be increased at lower water levels compared to higher. Water levels also exerted an effect on the microbial taxonomy, greater than the effect found with vegetation, with fungi showing a greater response than bacteria. These studies moved beyond some of our current analysis of relatively dry soils to community responses in wetlands with very high amounts of water. In a review by Dr. Naylor et al., “Trends in Microbial Community Composition and Function by Soil Depth”, the effects of both depth and moisture were examined through an analysis of previously published work [3]. This review detailed the critical difference between deeper soils and those near the surface. A better understanding of these deep soils will improve our modeling of the soil microbiome and its role in carbon cycling and greenhouse gas production, especially as, currently, surface soils are analyzed much more frequently than deep soils. Studies in this review showed that C stocks in deeper soils were more dynamic than those found at the surface, even if the rates of C cycling deeper in the soil were slower. Updating our models to incorporate microbiome activities in both deep and surface soil is thus critical to a fuller understanding of microbial activity in soil.

Keeping with the trend in examining soil types aside from those found inland, several studies presented here examined coastal soils and their effects on microbial systems. A study by Dr. Rodriguez et al., “Impact of Climate and Slope Aspects on the Composition of Soil Bacterial Communities Involved in Pedogenetic Processes along the Chilean Coastal Cordillera”, looked at the Chilean coast with a focus on four environments (arid, semi-arid, Mediterranean, and humid) and how these environments, along with resident microbial communities, affected pedogenesis (soil formation) [4]. They found that more stable environments (whether humid or arid) led to more microbial specialization. This study showed not only how soil could affect microbes but also how microbes could affect soil. Another study by Dr. Dong et al. looked at more saline soils of the coast (“Microbial Community Composition and Activity in Saline Soils of Coastal Agro–Ecosystems”) [5]. They found that salinity had little effect on bacterial richness but major effects on community composition and interconnectedness of microbial species. These studies will also be of great use for a better understanding of how microbial communities in soil may respond to shifting salt levels.

Moving away from native soils into more temporary environments, researchers at the Los Angeles Pierce College, led by Dr. Senn, explored how a wildfire changes both bacterial and fungal communities (“The Functional Biogeography of eDNA Metacommunities in the Post-Fire Landscape of the Angeles National Forest”) [6]. This analysis identified certain microbes that moved in quickly after a fire. The characteristics of post-fire resiliency for a microbe included photo-autotrophy, mineralization of pyrolyzed organic matter and aromatic/oily compounds, potential pathogenicity and parasitism, antimicrobials, and N-metabolism. As wildfires become more common, this work will help us better understand how the resident microbial community responds and who the first emergers are as the soil microbiome begins to revert to a more normal state. In “Landscape Composition and Soil Physical–Chemical Properties Drive the Assemblages of Bacteria and Fungi in Conventional Vegetable Fields”, Dr. Kumar et al. described microbial communities in intensively planted vegetable fields, an environment very different from native soil sites [7]. Their results showed that bulk soil (not immediately adjacent to plants) had a higher alpha diversity of both bacteria and fungi than rhizospheric (adjacent to plant roots) soil. These are important clues for the identification of key bacteria and fungi contributing to the plant–environment interactions and are of a practical significance for landscape-based ecological management. This will likely become of even greater importance as land tracts are either converted to agriculture or allowed to go fallow.

Much of the work that we do over the next several years will benefit from storing soil samples from certain sites, timepoints, or treatments. Air drying soil is one way to achieve this, but we do not know how air drying may affect the type of microbial information that we can extract from soil. Dr. Guo et al. in a recent study (“Environmental Difference and Spatial Distance Affect the Fidelity of Variation Source of Microbial Community Structure in Air-Dried Soils”) compared the fidelity of microbial analysis in air dried soils to fresh soils [8]. This work found a possible decline in explanation proportion when using air-dried soils to reveal microbial community patterns but also implied that air-dried soil is more suitable for addressing scientific questions under large spatial scales or environmental differences. These studies will be crucial for identifying when and how to air dry soils for long term storage before analysis.

Compost manure is often used to increase plant yields in agricultural systems. However, the effect of continued application of manure to soil microbiomes still needs study. In a new analysis, Dr. Dai and colleagues explored the role of predatory myxobacteria in different sites treated with manure (“Community Profile and Drivers of Predatory Myxobacteria under Different Compost Manures”) [9]. They found that the role of Mg^2+^ and Ca^2+^ concentrations had a major effect on the myxobacterial communities under different compost treatments. They also found that interactions between myxobacteria and other microbes (Micrococcales) were also affected as a function of manure treatment. Further analysis will show the long-term effects of manure treatment specifically as it drives inter-microbial food webs. Alongside manure, chemical fertilization is another method to increase plant yields. There are several different types of such fertilizers, and a study by Dr. Qiu and others (“Organic and Inorganic Amendments Shape Bacterial Indicator Communities That Can, In Turn, Promote Rice Yield”) showed how these amendments could both affect microbial community structure and rice yield [10]. They found that 10 bacterial taxa in the Actinobacteria phyla with roles in xenobiotic metabolism responded strongly to chemical fertilization. Modeling with a random forest approach reveled that these 10 bacterial indicator taxa acted as drivers for soil dehydrogenase, acid phosphatase, pH, TK, and C/N cycling.

A better understanding of how fertilization affects microbial communities or how air drying can change species presence and absence can both be explored through improved laboratory approaches. This can be helped though the use of synthetic soil testing systems, the development and analysis of which is explored in a paper by Dr. Smercina et al. (“Synthetic Soil Aggregates: Bioprinted Habitats for High-Throughput Microbial Metaphenomics”) [11]. These Synthetic Soil Aggregate (SSA) devices are 3D printed and provide a chemically defined and translucent environment that mimics soil systems. The authors show that these devices can be used to carry out analyses, including genomic, metabolomic, proteomic, lipidomic, and biogeochemical assays. They also show that a defined microbial community behaves differently in an SSA compared to in liquid growth culture. These SSA devices offer researchers the ability to obtain high-level data that show how spatial structure drives microbial ecology under a variety of environments.

The eleven papers published here represent some of the newest studies examining soil microbes in natural, agricultural, and laboratory systems. Much of this work answers critical questions related to soil microbiology, but, just as important, these experiments show us where to look next in our quest for a better understanding of how soil microbiomes affect ecological cycles on Earth.
